# A micro-costing analysis of nutritional support for persons with TB and their families in India

**DOI:** 10.5588/pha.22.0058

**Published:** 2023-06-21

**Authors:** G. Buonomo, C. Acuña-Villaorduna, V. Poongothai, M. Dharmalingam, C. Cintron, M. Dauphinais, S. P. Babu, L. M. Locks, S. Sarkar, P. Salgame, N. S. Hochberg, S. Lakshminaryanan, P. B. Narasimhan, P. Sinha

**Affiliations:** 1 Section of Infectious Diseases, Department of Medicine, Boston University School of Medicine, Boston, MA, USA; 2 Boston Medical Center, Boston, MA, USA; 3 Department of Preventive and Social Medicine, Jawaharlal Institute of Postgraduate Medical Education and Research, Puducherry, India; 4 Department of Health Sciences, Boston University College of Health and Rehabilitation Sciences: Sargent College, Boston, MA, USA; 5 Department of Global Health, Boston University School of Public Health, Boston, MA, USA; 6 Center for Emerging Pathogens, Department of Medicine, New Jersey Medical School, Rutgers Biomedical and Health Sciences, Newark, NJ, USA; 7 Department of Clinical Immunology, Jawaharlal Institute of Postgraduate Medical Education and Research, Puducherry, India

**Keywords:** TBI, nutrition, costing, India

## Abstract

Undernutrition is the leading risk factor for TB infection and death in India. We undertook a micro-costing analysis of a nutritional intervention for household contacts of people living with TB in Puducherry, India. We found that the total 6-month food cost for a family of four was USD4/day. We also identified several alternative regimens and cost-lowering strategies to encourage wider adoption of nutritional supplementation as a public health tool.

## Abstract (fr)

La sous-nutrition est le principal facteur de risque d’infection et de décès dus à la TB en Inde. Nous avons entrepris une analyse de micro-coût d’une intervention nutritionnelle destinée aux contacts familiaux des personnes atteintes de la TB à Puducherry, en Inde. Nous avons constaté que le coût total de la nourriture pendant 6 mois pour une famille de quatre personnes était de 4 USD par jour. Nous avons également identifié plusieurs régimes alternatifs et stratégies de réduction des coûts pour encourager une adoption plus large de la supplémentation nutritionnelle en tant qu’outil de santé publique.

The population-attributable fraction (PAF) of undernutrition on TB incidence in Puducherry, India is over 50%.^1^ Catastrophic costs of illness (>20% of annual income) can plunge persons living with TB (PLWTB) and their household contacts (HHCs) into food insecurity.^2,3^ Nutritional support may reduce TB mortality, decrease TB progression among HHCs and preserve human capital while being cost-effective.^2,4^ As nutritional supplementation is often perceived as cost-prohibitive, we present a micro-costing analysis of a nutritional intervention study in Puducherry to provide accurate data for policy research.

Tuberculosis: Learning the Impact of Nutrition (TB-LION) is a prospective nutritional intervention study based at the Jawaharlal Institute for Postgraduate Medical Education and Research (JIPMER) in Puducherry, an urban territory in Southern India (population: 877,000).^5^ TB-LION provides a 6-month nutritional intervention in which staff nutritionists visit undernourished, latent TB-positive participant households fortnightly to deliver rice, oil, groundnuts, multivitamins and three types of lentils to prevent TB progression. This comprehensive package, designed in consultation with local stakeholders, clinicians and dieticians, delivers 2,600 kcal/day for adults and 1,300 Kcal/d for children. The package contains enough of everything (except multivitamins) for all household members. At each biweekly visit, nutritionists weigh unused food, top off supplies and provide nutritional counselling. The estimated time devoted to counselling was included in our tally of hours worked.

We collected bottom-up cost data from a healthcare perspective at the JIPMER campus and during field visits in June 2022. We used the ingredients approach, wherein we identified resources necessary for the nutritional intervention (equipment, food, transportation and personnel time) by reviewing receipts and annual food vendor contracts, ascertained the cost of each component and estimated total intervention costs.^6^ First, we mapped the workflow of the nutritional intervention from the centralised procurement at JIPMER to final delivery to households. We then discussed with study personnel and observed—where possible—each step of the workflow to identify materials and personnel time utilised for different components of the process. As the study team pays no rent at JIPMER, we obtained rent estimates of comparable office spaces. We determined June 2022 market prices for items that were not purchased recently. We estimated personnel costs by multiplying their hourly wage (set by the Indian government) by hours worked. We assessed travel costs by multiplying distance travelled by the per-km rate charged by the transportation company which included vehicle, fuel and driver charges. We measured the distance and time required for travel during field visits. We calculated costs in Indian rupees (INR) and converted these to US dollars (USD) using the June 2022 weighted exchange rate (USD1 = INR78.015).

We divided costs into four types: fixed material, fixed personnel, marginal material and marginal personnel. The sum of fixed costs is the estimated cost required to initiate the nutritional intervention, and is not—to a point—dependent on the quantity of households. The sum of the marginal costs is the cost required for each additional household. We estimated the cost for a four-person household containing two adults and two children with all individuals receiving rations and multivitamins. We present overall costs associated with the intervention (Table) and breakdowns of cost components (Figure).

**TABLE:** Aggregated marginal and fixed cost for nutritional intervention*

Cost type	Cost components	6-month cost/household (INR)	6-month cost/household (USD)
Fixed material and overhead costs^†^	Rent, storage, measurement, and field supplies	3,354	43
Fixed personnel costs^†^	Personnel time for administration and intervention initiation	72	1
Marginal personnel costs	Nutritionist time	7,292	94
Travel costs	Driver and vehicle costs	12,740	163
Marginal equipment costs	Safe storage containers for recipients, personal protective equipment	1,390	19
Marginal food costs of TB LION regimen	Per package: 16.8 kg rice, 6.0 kg lentils, 0.26 L oil, 7.4 kg groundnuts, (2,600 Kcal, 82 g protein/person/day)^‡^	39,705	509
Marginal cost of two a day multivitamin tablets	1,456 multivitamin tablets	17,472	224
Total		82,025	1,051
Food costs of alternative supplementation strategies				
TB LION regimen without rice	Per package: 6.0 kg lentils, 0.26 L oil, 7.4 kg groundnuts, 112 multivitamins (1,300 Kcal, 59 g protein/person/day)^‡^	46,277	593
TB LION regimen without rice and half the groundnuts	Per package: 6.0 kg lentils, 0.26 L oil, 3.7 kg groundnuts,112 multivitamins (906 Kcal, 41 g protein/person/day)^‡^	37,637	482
TB LION regimen without rice, half the groundnuts and half the multivitamin capsules	Per package: 6.0 kg lentils, 0.26 L oil, 3.7 kg groundnuts, 56 multivitamins (906 Kcal, 41 g protein/person/day)^§^	28,901	370
TB LION regimen without rice, half the groundnuts and no multivitamins	Per package: 6.0 kg lentils, 0.26 L oil, 3.7 kg groundnuts (906 Kcal, 41 g protein/person/day)	20,165	258

*All costs were measured in and calculated for June 2022.

^†^Total fixed costs were divided by number of households in the intervention (*n* = 80).

^‡^All household members receive 2 multivitamin capsules per day.

^§^All household members receive 1 multivitamin capsules per day.

**FIGURE:**
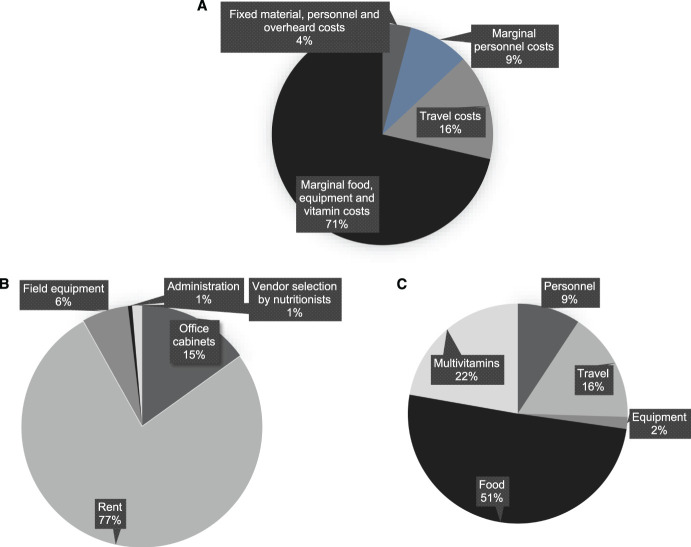
Breakdown of cost components: **A)** cost type; **B)** fixed cost type; **C)** marginal cost type.

We estimated the total fixed costs to be INR274,036 (USD3,513). We divided this number by 80 (the number of households receiving interventions) to get a per-household fixed cost of INR3,426 (USD44). This may be an artificially high number, however, as the equipment and personnel resources we observed in this study would be able to serve a higher number of households. The 6-month food and multivitamin cost per-family was INR57,177 (USD733) or USD4/day. Overall, the total per-household cost was INR82,025 (USD1,051).

Travel costs were a large component of overall cost. TB-LION recruits within a radius of 80 km so the 6-month travel costs could range from INR390 to INR21,844 (USD5–280). Reducing food delivery frequency to once a month could cut travel costs in half. Food could also be picked up at *anganwadis* (local community centres) or during TB clinic appointments which would essentially eliminate travel costs. As observed in a previous Indian TB cost analysis, personnel costs are considerable, making up almost half the marginal costs in the TB LION intervention.^7^

The biggest contributor to the cost was food. An Indian study showed that an intervention of only 1,089 kcal/d (including 41 g of protein) increased weight gain and TB treatment success rate.^8^ As the Indian government subsidises rice, groundnuts are often underused in the TB LION study, and multivitamins can be obtained inexpensively, we modified the TB LION intervention to formulate an alternative regimen (6.0 kg lentils, 0.26 L oil and 3.7 kg groundnuts per adult for 2 weeks). Despite food costs of only INR112 (USD1.4) per day per household, this regimen can provide 906 Kcal, including 41 g of protein, to adult recipients, which may be sufficient to improve nutrition and treatment outcomes.

Our analysis has limitations. The study obtained food at market prices and did not enjoy economies of scale. Furthermore, we assumed delivery of fixed rations for simplicity instead of topping off incompletely used supplies. Multivitamins were considerably more expensive than alternatives with similar nutritional value. These limitations inflated food costs. As we assessed costs over 2 contiguous weeks, we did not observe seasonal variations.

To our knowledge, this is the first micro-costing analysis of a nutritional intervention for TB in India. Although the TB-LION intervention may be cost-prohibitive in its current form, it is possible to deliver a pared-down version for as little as USD1.4/day to improve treatment outcomes and support PLWTB and their HHCs. The Indian government’s *Nikshay Poshan Yojana* provides INR500 (USD6.4) to PLWTB every month.^9^ This amount is insufficient for a substantial increase in caloric intake and cash transfers have been unreliable. Despite high costs, in-kind nutritional interventions may be cost-effective if they reduce undernutrition, TB mortality and TB progression among HHCs.^4^ We hope our study offers guidance to agencies interested in developing nutritional programmes.
